# Nursing Section

**Published:** 1904-01-23

**Authors:** 


### The Hospital.

### Nursing Section.

Contributions for this Section of "The Hospital" should be addressed to the Editor, "The Hospital" Nursing Section, 28 & 29 Southampton Street, Strand, London, W.C.

No. 904.—Vol. XXXV. *SATURDAY, JANUARY* 23, 1904.

### Notes on News from the Nursing World.

### THE MATRON OF OSBORNE HOUSE.

Miss Haines has been appointed matron of Osborne House, Isle of Wight, the King's Convalescent Home for officers of the Army and Navy. She has already taken up her duties. It will be remembered that Miss Haines was one of the two nurses who attended the King during his grave illness in 1902.

### ARMY NURSES ASKED TO VOLUNTEER FOR SERVICE.

Some excitement has been caused by the fact that a goodly number of sisters of the Army Nursing Service Reserve have lately been asked to volunteer "for service with the Army for not less than one year either at home, or in South Africa." Many of the sisters have responded to the call, but they are inquiring why it has been made. We understand that the chief reason is that nurses are required for temporary service in South Africa to take the place of some who desire, or need, to return to England. It may, perhaps, be as well to add that there are no apprehensions of any fresh outbreak of hostilities in South Africa.

### QUEEN ALEXANDRA'S IMPERIAL MILITARY NURSING SERVICE.

We are officially informed that Sisters F. M. Hall, E. T. Noble, R.R.C., H. F. Pocock, H. T. Young, and P. Young; also Staff Nurses M. M. Rees and L. F. A. Waller, have resigned their appointments in Queen Alexandra's Imperial Military Nursing Service. Miss A. L. Cox has been appointed matron and posted to Woolwich; Miss C. Mackay staff nurse and posted to Gosport; Miss E. Barber staff nurse and posted to Connaught Hospital, Aldershot. Sisters E. Beck, R. Osborne, and A. B. Wohlmann, and Staff Nurses S. B. Lanyon, C. G. Stronach, and E. M. Denne have been confirmed in their appointments; and Staff Nurse S. B. Lanyon has returned to Hounslow from the Royal Military College, Sandhurst.

### THE COLONIAL NURSING ASSOCIATION.

In response to a letter of appeal which has been issued by the Committee of the Colonial Nursing Association, the Goldsmiths' Company have subscribed £50 and the Mercers' Company 10 guineas. The ground of appeal is that a larger number of trained nurses is now required, necessitating an increased annual expenditure. We are glad to see some indication of a revival of the energy which brought the movement into existence. The more fully and constantly the public are kept informed of the progress of, and the need for, this valuable work in the colonies the more support the Association are likely to obtain.

### THE MATRON OF JOHANNESBURG HOSPITAL AND HER NURSES.

From further information which has reached us respecting the state of affairs at Johannesburg Hospital, we are satisfied that the decision of the board reported in our issue of November 28th, justifying the course the matron, Mrs. Magill, has pursued since she took up her appointment, was inevitable in the face of the evidence offered. It is a highly significant fact that although many of the nurses are well known to, and on friendly terms with—even being in some cases related to members of the board—these gentlemen were uncompromising in their verdict for the matron. With regard to the allegations of some of the nurses, one of them was to the effect that the matron insists upon a stretch of 12 months' night duty being done. We learn that the rule of the hospital regarding night duty is that "the nurses shall during their training perform night duty for at least six months, not necessarily concurrently." Another complaint was that she does not permit them to receive visitors in the nurses' quarters, whereas they may do so if they ask permission. It is absolutely denied by the authorities that the nurses' bedrooms are over-run by rats, and as to the alleged over-work of the night nurses, the average number of patients to each night nurse is 17, every nurse having a black boy to assist her with the rough work. As to the remaining complaint, that a large number of nurses are lodged in iron shanties, this appears to be true in reference to the night nurses, who occupy a temporary building of corrugated iron lined with wood. It is noteworthy that throughout the controversy the matron has observed a dignified silence. We do not doubt that under her judicious rule great improvements will be effected, and Johannesburg Hospital be brought into a line with leading English training schools.

### ROYAL BRITISH NURSES' ASSOCIATION.

A special general meeting of members of the Royal British Nurses' Association will be held at the rooms of the Medical Society, 11 Chandos Street, Cavendish Square, on Tuesday, February 2nd, at 4 p.m., in order to consider the draft of a Bill upon the State Registration of Trained Nurses, and any amendments that may be proposed. A resolution will be moved "That the Draft Bill, as amended, be approved; and that the Executive Committee be directed to take such steps as they may think necessary to have it submitted to Parliament."

### REGISTERED NURSES IN AMERICA.

A great deal has been said about the value of registration of nurses in America, but the United States Attorney-General has, in his official capacity, dissipated the idea that registration is essential in order to practise nursing in that country. In reply to a letter from the State Lunacy Commission asking whether the blank applications for registration which were sent out by the State Board of Regents to the State Hospitals need be filled in, the Attorney-General wrote, "The statute does not limit the right to practise nursing to registered nurses only; it does not require a Regents' certificate, or even a course of study in a training school for nurses, as a condition precedented to the right to practise nursing; it prescribes the conditions upon which one may obtain the right to the title of 'registered nurse,' but does not require unregistered nurses to discontinue their occupation. The statute merely prohibits persons who are not registered nurses from assuming the title, or using any abbreviation, word, letter, or figure to indicate that they are so registered." It is significant that the nurses in the State hospitals expressed themselves as satisfied with their present status, and declined to have anything to do with the blank applications for certificates of registration.

### THE VACANCY AT NORFOLK AND NORWICH HOSPITAL.

The explanation of the resignation of Miss B. C. Davis of the post of lady superintendent of Norfolk and Norwich Hospital, to which she was only appointed six months ago, is that she is about to be married. At the quarterly meeting of the Board of Management, it was stated that during that brief period, Miss Davis had, by unsparing attention to details of administration, successful training, and tactful management of the nurses, earned the esteem and gratitude of all with whom her duties had brought her in contact. As an indication of the efficiency of the nursing staff, it may be mentioned that in the Christmas quarter of 1903, Nurses Shand, Elgar, Gane, Braithwaite, and Carpenter were presented with medals and certificates, having completed their three years' training in the hospital, and passed satisfactory examinations. The first three were awarded honours certificates.

### MEALS IN A BEDROOM AT A WORKHOUSE INFIRMARY.

At the last meeting of the East Preston Board of Guardians a letter was read from Miss M. Sutton declining the appointment of nurse at the workhouse infirmary, offered her a fortnight ago. She stated that when she accepted the post she did not understand that she had to take her meals in a bedroom, nor that the bedroom she had to share was occupied night and day. It was affirmed by Major Andrews, chairman of the Nursing Committee, that Miss Sutton was fully conversant with the nature of the accommodation when she accepted the appointment. However that may have been, Major Andrews was obliged to admit that the bedroom was occupied by two nurses, one of whom slept in it during the night, and the other during the afternoon, and the earlier part of the evening. This is an absolutely indefensible arrangement, and no nurse who desires to maintain her health would consent to put up with it.

### BELFAST NURSES' HOME.

In the thirty-second annual report of the Belfast Nurses' Home and Training School, just issued, the letter of Miss Nightingale, which was read at the meeting in November, finds a place. It has already appeared in our columns. There are, we observe, from the statement of the lady superintendent, no fewer than 64 nurses now attached to the institution, which since September last when the Royal Victoria Hospital moved to its new premises, became a home for private nurses only. On the subject of private nursing generally, there is an admirable passage in the report pointing out that the private nurse learns self-reliance and adaptability in the larger world beyond hospital walls, and that she is able to put fresh courage into weary lives by the example of her own courage and by her own manner of life in the discharge of her profession. Miss Newman, the lady superintendent, is now, of course, free to give her undivided attention to the private nursing.

### THE IDEAL ASYLUM NURSE.

We congratulate the authorities of Leavesden Aslyum upon their new departure. They have issued this month the first number of their own magazine for the patients and officials. The idea is not novel, except for Leavesden, but it is admirably executed. The interior consists of the *Church Monthly*, while the cover affords space for notes upon passing events, notice by the medical and principal officers, and other items of interest to the community. There is one item of much interest to nurses. Miss E. M. Howell, the matron of Leavesden, having been asked to give a definition of the ideal asylum nurse, writes: "As so many of the patients admitted to the asylum suffer from bodily disease, I am strongly of opinion that an asylum nurse should have training both in an asylum and in a general hospital—say, one year in a hospital, and two in an asylum. The two experiences taken together would benefit both the nurses and the patients who came under their care. It would, too, be of considerable advantage to hospital nurses for them to have some training in an asylum. A hospital nurse coming to an asylum without any asylum experience is for some time 'at sea,' and, through no fault of her own, not so useful as she might be. The many little precautions absolutely necessary for the care of the insane can be learnt only in an asylum."

### DERELICTION OF DUTY AT A BRIGHTON HOSPITAL.

An inquiry before the Borough Coroner at Brighton into the circumstances of the sudden death of a child of three months old at the Royal Alexandra Hospital for Sick Children elicited information which quite justified the jury in adding to their verdict of death from natural causes, a rider to the effect that the house surgeon should be summoned at once in every case where serious symptoms were apparent. The evidence indicates that someone was at fault. When a nurse noticed, at about a quarter to six in the morning, serious symptoms about the child, she called the senior night nurse, and eventually the senior night nurse went for the sister. The sister asserts that when she arrived at 6.25 the child was dead, but neither she, nor the senior night nurse, summoned the house surgeon, though he was in the building within immediate call. The circumstance that the sister tried the use of restoratives for a quarter of an hour, in spite of her assertion that the child was dead, emphasises the fact that she took upon herself a responsibility which did not belong to her. The case, however, ought not to have been referred to her at all, but, as the house surgeon urged, the senior night nurse should have summoned him at once. If there is any rule which prevented her from taking this course, it should be abolished.

### FRICTION AT WEST HAM WORKHOUSE.

It may be necessary to ask the Local Government Board to hold an inquiry into the state of affairs at West Ham Workhouse. Four nurses have recently resigned their appointments; a nurse who seems to have incurred unpopularity at the workhouse has been removed to the infirmary; and the engagement of another, who had been in the service of the guardians for nine years, has been abruptly terminated, though it has been decided to give her a testimonial. Mr. Anderson, one of the guardians, affirms that it would be "a sorry day for some people," if the Local Government Board instituted an inquiry; but it is better that "some people," whoever they may be, should be disconcerted than that friction should continue to prevail, either at the workhouse or the infirmary.

### AN AGE QUESTION AT LEWES.

In sanctioning the appointment of the new superintendent nurse at Lewes Workhouse Infirmary, the Local Government Board called the attention of the Lewes guardians to the fact that on her appointment as superintendent nurse at a London workhouse infirmary in February, 1902, she stated that she was 30 years old, and that according to the details now given, she was still of the same age. We are not surprised that this discrepancy did not prevent the Local Government Board from approving the appointment, because there may perhaps have been a mistake in a figure, and the difference is of no practical importance. At the same time we, of course, strongly advise nurses to be strictly accurate in stating their age. Though we think that there should be no age limit for capable nurses, it is impossible to justify a departure from the truth, however apparently harmless. One of the Lewes guardians remarked in the case of the night nurse, that "she has got older in experience but not in age," and another that "some of the ladies move slowly;" but the principle at stake is too serious for banter.

### PROGRESS AT NEWPORT UNION INFIRMARY.

The Guardians of Newport Union in Monmouthshire have sanctioned an important scheme for the training of probationers in the infirmary. The medical officer and the superintendent nurse are to deliver between them a course of forty lectures to the probationers, to be designated "theoretical, practical, and midwifery," and in addition the superintendent nurse is to give "ward lectures" whenever it is convenient. The probationers will henceforth be required prior to the expiration of their period of training, to pass an examination by two independent examiners, and the granting of their certificates will be dependent upon the results of the examination. For the additional work imposed upon them the medical officer is to be paid half a guinea a lecture, and the superintendent nurse an honorarium of £10 per annum. It has also been decided to increase the nursing staff which will, in future, consist of the superintendent nurse, three charge nurses, eight probationers, and a male nurse. The inclusion of the latter is a step in the right direction.

### THE NEW INSTITUTE AT WELSHPOOL.

Miss Frances Howell, of Rhiewport Hall, formally opened the other day at Welshpool the new Nursing Institute, which has been erected by public subscription, on a site given by the Misses Howell as a memorial to the late Queen Victoria. The handsome gift was mentioned at the time it was made in our columns, and but for it, as Colonel Pryce-Jones, M.P., remarked in a speech at a luncheon given by Mr. Charles Howell prior to the opening, the commodious and necessary institute could not have been proceeded with, there being no other site available in the town. The institute is provided with two large wards, and a smaller one for the treatment of private patients; and there are a number of rooms for the nurses, the building being fitted throughout with the latest sanitary conveniences.

### THE IRISH NURSES' ASSOCIATION.

At a large and representative meeting of the Dublin Nurses' Club it was decided to change the name to the Irish Nurses' Association. During the course of the meeting Miss Kelly addressed the members on State Registration, and explained the scope of the rival Bills proposed to be introduced into Parliament. She urged Irish nurses to lose no time in appointing representatives to guard their interests, and keep them informed on the subject, pointing out that at present they are barely represented on either the Royal British Nurses' Association or the Society for the State Registration of Nurses. No resolution in the matter was, however, arrived at.

### THE NURSES OF TAUNTON HOSPITAL.

The distribution of prizes and certificates to the nurses of the Taunton and Somerset Hospital has just taken place. The awards were as follows:—Nursing: (1) Nurse C. M. Porter; (2) Nurse Conner; (3) Nurse H. Hicks. Anatomy: (1) Nurse H. Hicks; (2) Nurse C. Stainton; equal Nurses E. Yeoman and E. Stach. Physiology: (1) Nurse L. Stevens; (2) Nurse V. Newman; (3) Nurse H. Hicks; (4) Nurse C. Stainton; (5) Nurse G. Pilgrim. Very complimentary things were said by the Chairman, Major-General Emerson, of the manner in which Miss Bulteel, the matron, discharges her duties, and he frankly admitted that the abolition of the Nursing Institute Committee, which followed her advent, had not had the dire results anticipated.

### SHORT ITEMS.

The Court of Appeal has dismissed, with costs, an appeal against a judgment of the Vice-Chancellor of the County Palatine of Lancaster dismissing an action brought in order to restrain the carrying on of the Parkfield Nursing Home in the outskirts of Liverpool.—Miss E. H. Crewe, who has been matron of Crewe Isolation Hospital since its opening, has resigned her position.

### The Nursing Outlook.

"From magnanimity, all fear above;
From nobler recompense, above applause,
Which owes to man's short outlook all its charm."

### PRIVATE NURSING AND PATIENTS.

SIR WILLIAM BENNETT'S excellent address to the nurses at St. George's Hospital deals strongly with the relation between the private nurse and her patient, a relation which should rather be business-like than sentimental. Sir William refers with regret to the case of some nurses who wrote to a paper and complained "so few of our patients are grateful," and enlarged on their attachment for those they nursed and then grumbled that "it very seldom happens that a patient gives a nurse a present or leaves her a legacy." We have dealt with the question of gifts to nurses on this page before, and strongly expressed our disapproval of cadging by nurses and tipping by patients; now we would only deal with the whining nurse who thinks she is not appreciated, and that her work is so valuable that all her patients should leave her legacies. We have an uneasy feeling of having met this nurse, and having seen her kiss her patient, and heard her address her as "dear." As a nurse she was sloppy and untidy and seemed to be always running to sentiment; as the "handmaid" of the doctor she was neglectful and disloyal, and in her gush she always praised the nurse chiefly by dispraising the medical profession. It is a great pity she ever got her woes into print; the sensible and unselfish women who form the majority of the profession feel the disgrace keenly.

When a nurse goes to a private case she goes in her professional capacity, and as Miss Philippa Hicks once put it—"she must accept all kindness in the spirit it is intended, but bear in mind that gratitude tendered during danger and trouble is apt to diminish when the excitement of illness is over." In fact the nurse must not usurp the position of an intimate friend—it is destructive of her professional worth. It also hurts a nurse's position if she gives way to personal complaining and public grumbling. For "pessimism depends upon the overvaluing of self" and therefore betrays one of those melancholic and bilious creatures—

"Who seldom laugh and who never sing;
They do not join in the world's love chorus,
Themselves to themselves are everything."

And such people should rather be patients than professional nurses.

Surely there is no nurse who has been long in private work who hasn't learnt to take the rough with the smooth, and to make the good cancel out all the ill? If one case is very hard, another is very light; if one patient is very tiresome, another is a marvel of fortitude; if one patient is ungrateful, the next may be only too generous in both words and deeds. No nurse ever found the world "ungrateful" as a whole unless she herself was afflicted with ingratitude. So much depends upon the point of view. The active, conscientious, healthy worker is not bothered with "views" or what people think or say. She just does her work always to the utmost of her ability, and being a woman declines to see the world like a child through coloured spectacles. A true-hearted woman, who is also a nurse, cultivates a contented and impersonal spirit; then the trifling details of a tepid lunch or a hard pillow or a noisy bedroom will not worry us; even a rough-mannered doctor or a grumbling patient will only make us smile and wait quietly to see the other side of them; for there is good in everyone and sunshine everywhere, if we have only the grace to know and see.

But the nurse who frowns over her food, complains she cannot sleep, fusses about the way her bed is made, is in grievous error. She fancies she is the patient, that she needs all the attention, and that the house has to be run for her. It is no small part of the private nurse's training to learn to ignore these details and rise above trivial complaints. And it is merely a question of training with most of us to be able to sleep in any bed, to be able to eat whatever is offered us. Make up your mind not to worry over these things, to regard it as part of your work, and in a few months it will all be quite easy to you. At least it ought to be. It may happen that a nurse discovers that the strain of constantly re-adjusting herself to fresh conditions and companions is too contrary to her natural disposition to be pleasantly possible to her. Then she should seek some other branch of the profession. For Ruskin says we all ought to work, but also that all ought to be able to find joy in the working. To ensure that it is necessary that we should not ask too much of body or mind; and at the same time we should seek to have a healthy mind in a sound body. Then we should be free from sentimental grumblers, to the great benefit of our profession.

Sir William Bennett says that the refinement of character and the higher social status of the present day nurse make her relations with private patients more difficult. Sir William may be right as to the difficulty; but Carlyle shows that "great souls," *i.e.* refined characters, are "loyally submissive and reverent to what is over them," and no doubt find no such impediments to the pleasant and efficient discharge of the duties of a private nurse. "The higher social status" cult in these vulgar times is often but the outward sign of the "mean soul" within. We want womanly women as private nurses who, when they don their uniform, are well content to put the work first and to put off "the social status" carefully each morning before they start out to take their place as women workers in a busy world.

### Sir William Bennett on Modern Nursing in Private Practice.

(*Concluded from page* 217.)

PRIVATE NURSING AND THE THREE COURSES.

With regard to the actual commencement of private nursing, you each have the choice of three methods before you:—independent work, the association of yourself with some of the many co-operative institutions, and undertaking work in private nursing homes. Of these three it is probable, judging from the experience which I have had, that you will do well unless you possess some means of your own to attach yourself to one of the co-operative institutions, provided, of course, that you assure yourselves beforehand that the institution is one which has been well tried and is likely to be to your advantage. Independent work at first sight is attractive, but it is very prone to be intermittent, and in order to carry out your independent work well, you must of necessity attach yourselves more or less to a limited number of surgeons, physicians, or practitioners. So that the resources upon which the nurse has to rely are comparatively small, and in the event of the illness, or perhaps the death of any of those for whom she has been accustomed to work, she may find herself sometimes suddenly thrown into great straits, for it is by no means easy, if a patron is lost under these circumstances, to find another to take his place. In the case of association with one of the many co-operative institutions, you are as sure as you can be of continuous work; but of course there is this to be said in that respect, that you must be prepared at all times to undertake whatever is put before you, and you are constantly liable to serve a number of different superiors. So that in some respects the work may be less pleasant than it might be if you became successful in an independent position. But as I have already said the work is continuous, and so long as there is work to be had, supposing you attach yourself to one of these institutions you are sure to have a certain proportion of it, quite independently of what the work of any one or two or three individual practitioners may be. If, however, a nurse has some little income of her own, which will tide her over such times as she may be unoccupied, then independent nursing is in some respects the preferable course to adopt, provided of course that she is at the beginning sufficiently sure of the patronage of some medical men to whom she is known. The third alternative, the undertaking of work in private nursing homes, resolves itself very much into the same thing as attaching yourself to an ordinary hospital, with, of course, this great difference, that since the nursing homes are merely proprietary institutions they lack the stability of a long-established or well-known hospital, and of course the nurses connected with them do not participate in the prospects of a pension and other advantages which are shared by those who are able to associate themselves with an institution of a different kind, nevertheless, the life is pleasant; not altogether wanting in excitement whilst it is rarely monotonous. Taking all things into consideration I should advise the majority of you therefore who have to undertake the work of a private nurse, to put yourselves in connection with a properly constituted and well-known co-operative institution and to work from that.

ESSENTIAL QUALIFICATIONS OF A GOOD PRIVATE NURSE.

So far as the private nurse is concerned, it is necessary, if she is to be truly successful, that she should be possessed of certain qualities. It does not at all follow that a good hospital nurse—I mean a nurse who has proved herself an excellent officer in the course of her training—should later on turn out to be exceptionally good or even efficient as a private nurse. There are many people who, whilst they are excellent when working under supervision, are very indifferent when acting on their own account, and it is almost impossible, so far as I know, to tell with certainty—although frequently a pretty general idea can be formed as to the capacities of a nurse—whether a good hospital nurse will turn out to be a good nurse in private practice, mainly because the ordinary routine work in a well-conducted hospital provides little or no scope for the exercise of that tact and that power of accommodation to different circumstances and surroundings which is essential in the thoroughly competent private nurse. The embarking upon the career of a private nurse must therefore of necessity be something of an experiment, and you must bear in mind that the work in private is inclined to be monotonous. The excitement of operations, so stimulating in hospital work, is frequently wanting in private work; moreover, in an operation case, when the actual operation is over, there is the dull period which follows in watching the recovery and the convalescence. I have met with more nurses than one who are excellent at operations and for a certain time afterwards, but who, if the case is much prolonged subsequently become indifferent, and lose interest; in other words, become bored, and finally do not show themselves to advantage. In fact, there is a natural tendency with people of a certain disposition to tire of what is called a long case; persons of this type are not adapted for priving nursing. The essentials in the making of a good all-round private nurse are, in addition to the tact and facility of accommodation to different circumstances to which I have just alluded, steady level-headedness and good general average ability with an entire absence of tendency to excitement, to say nothing of that terrible thing in a nurse which is commonly called "gush." She should be sympathetic, but not morbidly so. And this question of sympathy between the nurse and the patient is a matter of much importance, for a reason which I shall mention shortly. Last, but not least, are two qualities which are absolutely necessary in the competent nurse—patience and gentleness, which together are met with only in those of good temper—another quality inseparable from good nursing. Impatience invariably begets roughness, which is an abomination in a nurse.

LOYALTY TO SURGEONS AND PHYSICIANS.

In private work, and also in hospital work, there is one point which should never be absent from your minds, and that is the absolute necessity for loyalty to the surgeons, physicians and practitioners under whom you work. If there is any lack of this loyalty it is most unfortunate not only for the patients but for all concerned. You must understand that a nurse in a vast number of cases is in a position in which she can influence the patient to a very large extent; and a hint thrown out under certain circumstances may undermine the patient's faith in a medical attendant to a degree which may prove disastrous not only for the medical attendant, but for the patient, and generally also to the nurse. It should be borne in mind that the recovery of patients, more frequently than not, depends as much upon their confidence in their doctor as upon anything else; and sometimes the recovery depends absolutely upon that faith. If by any incautious word or any intentional remark the confidence of a patient is shaken in the doctor, the recovery of that patient and the issue of that case may frequently be unsatisfactory if nothing worse. I could give you instances in which a very slight remark has led to very uncomfortable complications. I remember on one occasion having operated upon a patient for a condition which, under ordinary circumstances, was easily and rapidly curable, but which in this particular case was extremely difficult, in consequence of previous neglect, a prolonged period before complete recovery therefore being probable. That being the case, I told the patient that it would be necessary to keep in bed for at least four or five weeks. The nurse who was looking on said, " Dear me! how sad. The last case which I had of this kind, which was operated upon by Mr. So-and-so (mentioning another surgeon) was up and well in ten days." You can easily imagine what the effect of this remark was upon the patient; and what the effect of it might have been upon my professional relations with the patient, had they been less assured than they fortunately happened to be.

### The Question of Presents.

A very important question in the matter of private nursing is the general relation of the nurse to her patient. Almost as I was thinking over what I had to say on this subject, which is one which I had intended dealing with, there appeared in a weekly paper (*Truth*, December 17th) a letter from a nurse, which I will read to you, because, from an unpleasant aspect, it is interesting:—

"Sir,—I have been a nurse for over ten years, and during that period I have attended many patients, some rich, some poor, some who were more or less alone in the world, and all of them very sorry for themselves. That I write this letter to you is the result of a conversation which occurred at our institute this week. In comparing our experiences on this occasion, it surprised all the nurses who were present that so few of our patients are grateful. They pay us the small weekly fee much as they pay the wages due to their servants, but they expect us to be as interested in their case as if we were members of the family. It very seldom happens that a patient gives a nurse some small present, remembers her when she has left, or leaves her a legacy.

"We often become much attached to a patient, which is easily understood, as we frequently are months with them, during which time we are generally together, and many of them talk without restraint of themselves and of their affairs. When they can dispense with our services they pay us our last fee, thank us more or less warmly for our care and kindness, and, having parted with us never even write a line either at Christmas or at any other time. Yet some of those patients owe their lives more to our careful nursing and exertions than they do to the doctor and his medicines. Many a nurse does a hundred and one things for her patient which it is no part of her duty to do, and most of them are keenly anxious to bring the case to a favourable termination —as anxious, indeed, as any member of the patient's family. Having talked the matter over, I was asked to write this letter to you, hoping that you might comment upon it.—A Nurse."

Nothing could well be more deplorable than a letter of that kind, and it is in my opinion to be regretted for the sake of nurses generally, that any paper should have thought fit to publish it, and further to comment upon it favourably. It must be understood—and you cannot let this sink too deeply into your minds—that the mental condition of sick people is abnormal, and that sick people will say and do things which people who are healthy would not think of saying or doing for a moment. So that you will not infrequently find that in your relation of nurse to a sick person things will be said to you, promises may be made, all sorts of ideas may perhaps be suggested to you which would never for one single moment be thought of by a person in a normal condition of body and mind. Do not therefore regard too seriously promises or undertakings of any kind made or suggested to you in the capacity of nurse by patients when they are very ill, and do not attach too much importance to apparent friendships made under the same circumstances. Patients under those conditions often lose their sense of proportion and are not quite responsible sometimes for what they say, although so far as you can see they may be perfectly rational and be able to converse upon and deal with the ordinary affairs of life.

### The Wrong Spirit.

In this letter which I have read to you, complaint, as you will see, is made by a nurse of the general want of gratitude in patients after they have recovered from their illnesses. Now, first of all, bear in mind the point that I have just mentioned, that the peculiar mental condition of sick people leads them sometimes to say things, to do things, to contract apparent friendships, which are practically impossible under normal conditions. This being the case, if you pay too much attention to what is said and done under these abnormal conditions, you may be led to expect, after the patient's recovery, that things may happen to you and that things may be done for you which as a rule certainly will not take place. But the worst part of the letter is the spirit in which it is written. You will notice that the complaint of the nurse is that the patients show no gratitude for what is done, and she complains that they merely pay the "small weekly fee" which she contracts to serve them for, and which by common consent is considered fair and that they do nothing more. In other words, during the time she is working for a remuneration the amount of which she well knows at the commencement of her engagement, she has in her mind the possibility of something further coming from the case afterwards. Believe me this is altogether the wrong spirit with which your work should be performed. I cannot imagine anything which could reflect more discredit upon the calling of a nurse than a letter like that. My experience is that in nearly all the cases in which the services of a nurse seem not to be appreciated, or in which no special mark of gratitude is conferred that the fault (generally want of tact) lies with the nurse and not with the patient. In connection with this subject it may interest you to know that there is a small body of nurses working in private practice in London who are under an obligation, which they rigidly observe, to take no present or reward of any kind from a patient beyond their legitimate remuneration. I think this is carrying a principle unnecessarily far, but the spirit which prompts it is the true one which should dominate you in your work. My own view is that if a nurse is offered a present, whatever its form may be, that she is right in accepting it, but it will be a grievous day for the calling of a nurse if the public are led to think that there is expected as a matter of course some mark of "gratitude" at the end of her engagement. No! Remember there are such things as respect for your calling and loyalty to yourselves, and it is only by the exercise of that loyalty that you will maintain your profession in the honourable position which it should occupy.

### Queen Alexandra's Imperial Military Nursing Service.

### SISTERS' AUTHORITY AND TRAINING OF ORDERLIES.

NEW standing orders for the Royal Army Medical Corps and Queen Alexandra's Imperial Military Nursing Service have just been issued, and contain some notable additions. They assign a definite position to the sisters for the first time. Sisters and orderlies are no longer to share the work between them, but the latter will act under the immediate orders of the sister, and, in the words of Paragraph 296, "They will in hospitals, when working under sisters and staff nurses, give prompt obedience to all instructions given to them." Orderlies can now obtain a three years' training with lectures and instructions from the medical officers, matron, and sisters, together with a certificate after duly passing the needful examinations, and thus become qualified nurses.

### A SISTER-INSTRUCTOR FOR THE ORDERLIES.

The nursing recruit is to be attached to the wards and to be under the immediate instruction of the sister and staff nurse. The sister in charge is to teach the practical management of wards, the nursing of helpless patients, adminstration of medicines, application of external remedies, and the preparation of various diet drinks. The matron will satisfy herself that the instruction given by the sisters is in accordance with the syllabus and is given daily in a systematic manner, and she will detail a sister to assist in the examination of the recruits, and will herself take part in the final examination. During the two months' preliminary training at Aldershot the sister-instructor will hold classes and give demonstration amongst others on the following subjects:—Personal cleanliness, cleanliness of the ward, cleanliness and disinfection of all utensils, bed-making, correct reading of measure glasses, use of the clinical thermometer, registration of pulse and respiration, padding of splints, and making and applying bandages. An elementary course of eight lectures on anatomy, physiology, and first aid will be given by the medical officer. The sister-instructor will attend these, and then hold classes of not more than six men to explain what they do not understand and to help them generally.

### THE THREE YEARS' TRAINING.

Instruction for third-class orderlies is given by the matron, and includes besides further details of the subjects mentioned above, methods of reducing temperature, different sorts of baths, enemata, fomentations, gargles, injections, eye-drops, application of ice; nursing of enteric, pneumonia, pleurisy, paralysis, apoplexy, phthisis, and acute rheumatism, surgical dressings, and general observation of patient. The second-class orderlies are instructed in the qualifications of a good surgical nurse; wounds and their antiseptic treatment, burns and scalds, fractures, bleedings, tracheotomy, joint and head, and abdomen injuries and the preparation for, and after treatment of, operations. They also have lectures given to them on the principles of diet, the nursing of heart cases, the lungs and respiration, the nursing of skin diseases and tubercle cases, infection and disinfection. In all there are twelve lectures on medical, and twelve on surgical, nursing. The first-class orderlies as well as special lectures on the nursing of enteric fever, dysentery, dengue, Malta fever, malaria and cholera, are taught the care of instruments, the management of operating-rooms, massage, chiropody, and advanced sick cookery. After completing one year's service as third-class orderly, and passing the necessary examination, an orderly will be promoted to the second-class, and a second examination is needed at the end of the second year to become a first-class orderly. No orderly will be recommended for advancement unless he has uniform good reports from the officer and sister of his ward. A certificate of training, signed by the principal medical officer, matron, and examiner, will be given to the orderly if he passes his final examination successfully after the three years' training.

### THE POSITION OF SISTER.

In hospitals where a matron and nursing staff are employed, the wardmaster is still responsible for discipline, but he is to be "careful not to interfere with the duties assigned to the matron and nursing staff." He is to hand over to the sisters any stimulants required for patients in the wards. In wards nursed by sisters the duties laid down for the wardmaster will be performed by the sister in charge. In any cases of doubt or difficulty the ward orderlies are to refer at once to the sister or non-commissioned officer, to whom they are also responsible for such articles of equipment and bedding as may be handed over to them. They may not leave their wards except at the hour stated on their time-tables without permission of the sister or non-commissioned officer in charge of the ward. In the event of any irregularity or sudden illness in the ward they are to report immediately to the sister or non-commissioned officer. A private is not eligible for appointment as lance-corporal or promotion to corporal's rank if he belong to the nursing section unless he has passed through the course of instruction for second-class orderlies and obtained satisfactory reports of his work from the ward sisters.

### The Application of Hot Irrigation to a Leg with a Compound Fracture.

### EXAMINATION QUESTIONS FOR NURSES.

THE question was as follows:—If ordered to apply constant hot irrigation to a leg with a compound fracture, in a cottage where appliances were limited, how would you proceed?

### ONLY ONE PRIZE THIS MONTH.

I regret to say that with one exception the papers are none of them good enough to merit prizes. The question has not been sufficiently thought out and the connection of cause and effect not been studied, whereas some of the competitors show a lamentable ignorance of the simple engineering laws, necessary for the carrying out of so many things in our daily life. It was chiefly to help nurses to apply their common sense, their powers of observation, and their knowledge of the relations of cause and effect to the circumstances of their daily professional life that these examinations were started and though of late there has been a marked improvement in the answers received, there is still much to be desired. In such a case as that suggested in the November question, a nurse would almost certainly have to arrange the whole thing herself. Generally the doctor orders the treatment, but leaves the nurse to execute her ingenuity in carrying it out, and it seems probable that he would be far from pleased on viewing the arrangements proposed by some of our nurses.

ONLY ONE REALLY SATISFACTORY ANSWER.

This is sent in by "Aubrey," whose paper is admirable, practical, careful and comprehensive. But a prize is lost by one little oversight. The author omits to explain how and why a funnel should be placed in the upper vessel, nor is there any description of the manner of its fastening. It will be desirable to have this question again before long, and we must hope that the general results will be better.

DEFECTIVE PAPERS.

"Jessica" begins by saying she should seek the doctor's permission to have both the patient's legs outside the bed on a chair. Compound fractures are not to be thus lightly treated!

"Lady Betty" says, "first obtain three strong helpers (men if possible)." In a cottage it is fortunate if one strong helper is to be found.

"Nurse F." sends a careful paper, but her arrangements are not simple enough to be carried out satisfactorily in the presumed circumstances.

"R. J. R." proposes to pour the irrigating liquid from a jug. This mode is not that of "constant irrigation."

No one has sufficiently considered the difficulty of keeping the liquid at a stated temperature except "Lady Betty," and her paraffin stove might be difficult to obtain, and when found prove very dangerous.

QUESTION FOR JANUARY.

Describe the arranging, preparation, and application of an abdominal fomentation. THE EXAMINER.

RULES.

The competition is open to all. Answers must not exceed 500 words, and must be written on one side of the paper only. The pseudonym, as well as the proper name and address, must be written on the *same* paper, and *not on a separate sheet.* Papers may be sent in for fifteen days only from the day of the publication of the question. All illustrations strictly prohibited. Failure to comply with these rules will disqualify the candidate for competition. Prizes will be awarded for the two best answers. Papers to be sent to "The Editor," with "Examination" written on the left-hand corner of the envelope.

N.B.—The decision of the examiner is final, and no correspondence on the subject can be entertained.

In addition to two prizes honourable mention cards will be awarded to those who have sent in exceptionally good papers.

### A Function at the North-Eastern Hospital for Children.

THE North-Eastern Hospital for Children, Hackney Road, has recently, owing to readjustment of boundaries, been included in the Borough of Bethnal Green, and on Monday it was visited by the Mayor and Councillors in State. A number of ladies were also present, and the guests were received by Miss Bushby, the lady superintendent, Mr. Glenton-Kerr, secretary, the Rev. W. G. Morcom, hon. chaplain, and several members of the committee. Mr. Walter Johnson, J.P., welcomed the Mayor, and said that the hospital had grown from very small beginnings to its present size, and that the four wards just added were occupied within a week of their completion. There were now 114 beds, and there would be an additional cost of £3,000 per annum for upkeep, *i.e.*, £9,500 had to be provided every year. There were over 1,300 attendances of children every week. The presence of the Mayor was, he added, very cheering because it proved the interest taken by the locality. The hospital was the largest in that part of the metropolis and the second largest in London. The Mayor, Mr. Charles Wood, J.P., having replied, assuring the company of the deep interest felt in the hospital's welfare by the members of the Borough Council, a tour was made of the new buildings. The additions include four wards of 11 beds each, an ophthalmic and an isolation ward, a theatre, an anæsthetising room, and a pathological laboratory. Among the arrangements specially welcomed by the nursing staff are the spacious kitchens attached to each ward, the tiled ward lavatories, with a special flush-fountain spray; the electric lights which can be detached from the wall and carried to the bedside; and the Lawson-Tait cots, with easily-sliding sides, which simplify the process of bed-making. Tea was served after the inspection by the lady superintendent and the home sister.

### St. Bartholomew's Hospital, Rochester.

OPENING OF THE NURSES' HOME. BY A CORRESPONDENT.

THURSDAY, last week, was a red-letter day in the history of this, the oldest hospital in the world, for it witnessed the opening of the new Nurses' Home. When the famous Gundulf came to England with the Conqueror he built the Norman portions of Windsor Castle, the Tower of London, Rochester Cathedral and Castle, and founded a hospital for lepers, with a prior and four brethren for the staff. In the early part of the fourteenth century sisters were added. The rapacious Tudor, holding that a hospital was a "superstitious use," tried to seize the endowments. In this he did not altogether succeed, but dark days fell on the community. The original Norman chapel, built in 1078, continued to exist though sadly desecrated, and formed a centre round which the property cohered, until in 1863, through the agency of the then Dean of Rochester, the institution blossomed out into a general hospital. After a renewed life of 40 years, it has now put itself into line with the very best of the minor hospitals at an outlay of £14,000. Of this amount the munificent sum of £5,500 was contributed by Mr. Thomas Hellyar Foord, of Botley Grange, Hants, for the building and furnishing of a nurses' home. The handsome edifice contains separate bedrooms for five sisters and 21 nurses, with bath-rooms, etc., on every floor. There are large and commodious sitting-rooms for the sisters and nurses respectively, and a workroom with sewing-machine has been thoughtfully provided. Box-rooms, bicycle-rooms, and everything to ensure comfort have been remembered. While there is a fireplace in every room, the corridors are heated by hot water, and the building is lighted by electricity throughout. The same spirit of generosity and forethought has been manifested in the furnishing. The polished floors of the bedrooms are laid with Oriental rugs, and each room has a neat writing-table and comfortable easy chair, beside the usual furniture. The sitting-rooms are equipped with a goodly selection of Chesterfield sofas, easy chairs, occasional tables, and all that goes to make a room look homely. No reasonable expense has been spared in the endeavour to get furniture which will not only serve its present purpose, but will prove enduring. A suitable dedication service was conducted by the venerable Dean of Rochester, who is Patron of the Charity, and was attended by the Mayors of Rochester, Chatham, and Gillingham, and in the evening there was a pleasant reception and dance by way of house-warming. The space in the main building vacated by the nurses will be converted into a much-needed children's ward, and the hospital will then have 100 beds.

### Everybody's Opinion.

[Correspondence on all subjects is invited, but we cannot in any way be responsible for the opinions expressed by our correspondents. No communication can be entertained if the name and address of the correspondent are not given as a guarantee of good faith, but not necessarily for publication. All correspondents should write on one side of the paper only.]

### STATIONARY SISTERS.

"HOSPITAL SISTER" writes: May I be permitted a few words on the above subject from a "Sister's" point of view. A nurse by the time she has completed her training and is sufficiently experienced to be a ward sister, has usually found out the particular branch of hospital nursing which she prefers and for which she is best fitted, and applies for a post as head of a "male accident," "children's medical," "gynæcological," etc, as the case may be. She has no wish to be a general factotum, "to serve the institution as required," any more than has the medical man who applies for appointment as "physician," "surgeon," "obstetrician," "ophthalmic surgeon," "pathologist," etc., and she should be equally free to choose her own line of work. As ward sister she soon becomes thoroughly acquainted with her physician's or surgeon's methods and "ways," takes great pride in the thoroughness of her work, training of her nurses, and management of "her ward and her patients," which laudable pride, I think, "Matron" is a little hasty and unkind in describing as "egotism." There are disadvantages in the "stationary" system no doubt. The one that "Matron" fears is that the sister may become a "personage," but that is the way of clever hard-working people with a definite object in life in whatever profession they are found. They are apt to rise from a dead level of uniformity and do not remain mere cyphers to "serve as required." It is a real drawback that a permanent staff of sisters blocks promotion. One large general hospital which always trains its own sisters has no less than seven out of twelve ward sisters who have been in the same wards for from seven to over twenty years. There is, therefore, very little chance of promotion for its graduate nurses to the rank of sister in their own training school. I would suggest that the appointment as sister of a ward should be for three years only, and not practically for life, as it very often is now.

"A MATRON" writes: Having read your note on this subject, I should like to give my personal experience of both sides of the question. I was trained for four years at one of the larger hospitals in London, where there were stationary sisters. By good luck for the last six months I was put on as "holiday" sister, and went through most of the principal wards. Had it not been for this circumstance, I should have come away with a very imperfect knowledge of many branches of work. My time had been mostly spent in male wards where the sisters had been for over 12 years, and absolutely nothing was taught me of female nursing. The sisters were excellent over male wards, but were settled in one groove. I worked for over eight years in London, either under a stationary sister or as one, and do not uphold the system. I have now been matron for seven years, and my sisters are not stationary. I find it a great advantage both to myself and to them. Suppose sister No. 1, whose wards have a great many anxious cases, is taken ill suddenly, or is telegraphed for to go home. I look round and see Sister No. 2, who was in charge of No. 1 wards a few months ago, has very light cases, nothing that a senior nurse cannot manage. I promptly move No. 2 to No. 1 wards. She knows the doctor's ways and how the wards are worked, and it only takes her a day to pick up the threads and learn up her cases. It is an immense relief to a matron to be able to do this. It is a great advantage to a sister to be kept constantly educated in the different branches. Look how the ways of surgery are changing; almost every six months something fresh is being found out and new ways of conducting operations. I venture to say that if a sister went back to the operating theatre after two years' absence she would find herself "old fashioned," unless she had kept herself very well informed of the advances made. Doctors, I think, might be opposed to the changing system. It is pleasant to have someone that knows one's ways. At the same time, I have always found that if doctors found a new nurse anxious to learn, they were very patient in teaching her. The disadvantages are so small compared with the gain that they are hardly worth mentioning. I find that my linen stock is not so well looked after and the wards are not made so pretty. A sister will not take the trouble to collect the number of nice things, if she is leaving it in six months. Still these are small drawbacks compared to the great advantages a sister gains in being so "at home" in every branch of her work, that no matter what emergencies arise, she is so thoroughly sure of herself, that she is never at a loss as to what she should do. It calls forth a spirit of friendly rivalry which, I think, does good. No sister likes to think that the former one kept the wards better. It is also better training for any sister who looks forward to being a matron. I have known night superintendents who were drawn from the ranks of medical stationary sisters, who were quite unfitted to take charge of the serious operations that often take place during the night. I hope your note may induce matrons to give it a trial.

### ASSISTANT NURSES IN WORKHOUSE INFIRMARIES.

"A POOR LAW MATRON" writes: In reply to Miss Annie Louisa Lee's letter, I beg to state, with all due respect to the Meath Workhouse Nursing Association which has done excellent work in the past, that in at least some of the largest Poor-law infirmaries the days of the assistant nurse are past. Though allowed by the Local Government Board to have some 18 assistants in the original order for this infirmary of 700 beds, there are none, but an increase has taken place in the number of sisters and probationers—the probationers in their third year acting as staff nurses. The vacancies for sisters or charge nurses are filled up by the most efficient of the probationers when their time is completed, and from other hospitals and infirmaries, great care being taken to select those who are thorough nurses, as well as capable women for training probationers. For this infirmary—the largest in the South of England—I may say the supply meets the demand, with a sprinkling of outsiders which is always desirable. I may add that where possible preference is given to a candidate who, after her three years here, can take a little time elsewhere—and return—so as to increase her experience and expand her mind, and that of the nurses who leave and go on with their profession quite a third remain nursing under the Poor-law in various positions. In these days when efficient nurses are in such demand it seems a pity to advise a candidate to go in for less than three years' training.

### DISTRICT NURSES AND PATIENTS' PAYMENTS.

"EX-QUEEN'S NURSE" writes: I should be glad to have the opinion of other nurses—especially "District" and "Queen's"—on the matter of patients' payments for the services of a district nurse. It seems to me a bad plan to make these entirely free, as is nearly always the case—only giving the patients the option of sending a donation if they feel inclined. Unless these donations are personally asked for and collected—not by the nurse of course—they are far more often forgotten or indefinitely postponed than not. This has been my experience, and not from any ingratitude either, but simply from slackness or the dislike of taking sixpence or a shilling—often quite as much as the giver could afford at once—to the treasurer, some important person perhaps of whom they stand in awe. Now, why should not the district nursing be worked on the same principle as a medical club? each family who wished to have a right to the nurse's services paying, let us say, 3d. monthly. This might be done in well-regulated parishes through the district visitor. If 150 families in the place did this, it would bring in annually £22 10s., or about a quarter of the sum needed to support the nurse. Perhaps there would not be nearly so many families able and willing to do this—but at any rate in any small town, there should be no difficulty in finding some. Those who did not pay and needed the nurse might give, say 3d. a week, the two first visits being always free. There would necessarily always be many entirely free cases; these I think should be left to the discretion of the doctor and nurse, who would represent them to the committee. People in better circumstances—not subscribers—would be expected to give more than the 3d. weekly. Of course thankofferings might still be made, and in five years' time the Association might easily become self-supporting; indeed, it would be as well to warn the people that if it did not, some alterations might have to be made. The alms of the well-to-do will no doubt always be required to launch the association and make up deficiencies, but to let the people know that the rich are paying entirely for them seems to me a form of pauperising in any average district. I am not speaking of the extremely poor. Would the people not value the benefits they received more if they helped a little, and would it not be more conducive to self-respect? In an entirely new association where they did not know the advantages of having a nurse this system might not be practicable at first, but in a couple of years it should become so.

### The Nurses' Bookshelf.

THE HOME NURSE. By S. F. A. CAULFIELD. (London: Elliot, Stock. 171 pp. 3s. 6d. net)

THE authoress of "The Home Nurse" tells us she is not a trained nurse, though she has had much experience of sick nursing in private houses. There is much useful information to the home nurse, especially in the chapters on "ordering of the sick-room," and "essential rules." But it is a mistake to give instructions to an amateur as to the administration of a steam bath, and there should have been a little more care in the wording. We feel sure that the intention was not to advise the nurse to leave her patient naked except for a covering to the calves of his legs, but that is the impression conveyed. The advice to pour from 3 to 5 gallons of cold water over a sick person sitting on a stool in a large tub is certainly not good. There are some useful hints in the chapter concerning external applications, especially those relating to the fomentation kept hot by a stomach tin. The suggestion as to the usefulness of pine-apple juice in diphtheria is interesting, and we believe novel in this country; it is worth remembering in an emergency. The juice of the pine apple might act beneficially on the tough membrane which forms across the throat in that fell disease. Chapters XVIII. and XIX. are in doubtful taste.

PREACHING AND HEALING. By Dr. HERBERT LANKESTER. (Published by Church Missionary House, London. 1903)

THIS interesting book is the report of the C.M.S. Medical Mission Auxiliary for the year 1902-03. It contains the reports of the annual meeting, of the medical committee, and of the various mission stations in Africa, Asia, and North-West Canada, together with a statement of contributions to the mission for the financial year (April 1st, 1902, to March 31st, 1903). We regret to observe that although during this time the income has increased 30 per cent. the mission is in debt to the parent society for no less a sum than £3,270. The maps of the Mohammedan lands in the East and of China and Japan, greatly add to the value of the book, a careful perusal of which should convince any thinking person of the immense amount of practical good, both at home and abroad, performed by the doctors and nurses supported by this mission.

FOR HER SEX. Translated from the German. (London, William Heinemann.

NOVEL readers in England have recently been deluged with books dealing with sex-problems. It is interesting to note that this sudden awakening to the responsibilities of women with regard to handing down to their possible offspring a heritage of disease and vice, by an injudicious and selfish choice in marriage, has spread even to Germany. I say "even" to Germany, because although in most spheres of intellectual activity Germany is at least on a level with any other European country, yet in the matter of the equality of the sexes, man's supremacy has hitherto remained almost unchallenged in the Fatherland. "For Her Sex" is the anonymous diary of a German girl, who is deeply in love with a man whose past life she suddenly realises to have been such as to render him unworthy of her trust and respect. The struggle of her better nature with her very human love for the sinner is extremely painful reading. We cannot go behind the statement in the preface that this is the poignant record of a personal experience, but even if the "diary" should be after all only a literary device, at least the author has thought deeply and felt deeply with regard to the painful problems which the modern woman has dared to face. The impression the pages convey is that the girl, by whom they claim to have been written, hoped that the publication of her own fruitless struggle would enable others to take up the burden of solving a question for which she had neither physical nor moral strength. The soul-struggle overwhelmed her, and she found the very Pagan solution of her troubles in suicide, leaving the ethical problem yet unsolved. The little book has gone through ten editions in Germany, and is now translated for the first time into English.

### Appointments.

**[No charge is made for announcements under this head, and we are always glad to receive, and publish, appointments. The information to insure accuracy should be sent from the nurses themselves, and we cannot undertake to correct official announcements which may happen to be inaccurate. It is essential that in all cases the school of training should be given.]**

BRISTOL ROYAL INFIRMARY.—Miss Florence A. Pitman has been appointed midwifery sister. She was trained at Guy's Hospital, London. She has since been engaged in private nursing in connection with Guy's Nursing Institution. She holds the L.O.S. certificate.

PONTYPRIDD INFIRMARY.—Miss F. A. Foulds has been appointed charge nurse. She was trained at Southwark Infirmary, London, and has since been charge nurse at Toxteth Infirmary, Liverpool, and staff nurse at the Mountain Ash Accident Hospital.

WOLVERHAMPTON GENERAL HOSPITAL.—Miss Ellen Gertrude Woodward has been appointed night sister, and Miss Sybil Hughes Hallett has been appointed sister of fever wards. Miss Woodward was trained at Guy's Hospital, London, and has since been sister at Royal Hants County Hospital, Winchester, sister at Essex and Colchester Hospital, and sister at the Royal Infirmary, Halifax. Miss Hughes Hallett was trained at Addenbrooke's Hospital, Cambridge, where she has since done staff nurse's work. She has also done service as staff nurse in Queen Alexandra's Imperial Military Nursing Service. She holds the L.O.S. certificate.

### Presentations.

DEACONESS HOSPITAL, EDINBURGH.—Miss Rachel C. M. Reid, who has been for nearly three years sister in the Deaconess Hospital, Edinburgh, has been presented by the surgical and medical staff of the hospital with a handsome dressing-case with silver-mounted fittings, and by the nursing staff with a solid silver antique teapot, on the occasion of her leaving for the post of matron of the Cottage Hospital, Axminster.

### Echoes from the Outside World.

### Movements of Royalty.

The King arrived at Buckingham Palace from Sandringham between six and seven on Monday evening. The Queen travelled to town on Wednesday. Both their Majesties, with several other members of the Royal family, will be present at the service at Windsor on Friday in memory of Queen Victoria.

### The Wedding of Princess Alice of Albany.

The bridesmaids at the wedding of Princess Alice of Albany and Prince Alexander of Teck on the 10th of next month will be Princess Victoria of Wales, the Princesses Margaret and Patricia of Connaught, and the Ladies Alexandra and Maud Duff, daughters of the Duke and Duchess of Fife. Prince Francis of Teck will be his brother's best man. The gift of the King and Queen will be a service of silver plate. The ceremonial at the wedding will be similar to that on the occasion of the marriage of the Duke and Duchess of Albany in 1882. The guests at Windsor Castle will drive to St. George's Chapel down Castle Hill, through Henry VIII's. Gateway to the Horse-shoe Cloisters, and enter the chapel by a covered way at the west door. The Archbishop of Canterbury will officiate, and he will be assisted by several bishops. After the ceremony the King and Queen and the Royal family will lunch in the State dining-room, luncheon being provided for the guests in the Waterloo Chamber.

### The Czar and Peace.

On the occasion of the Russian New Year last week the Czar held a reception of the Diplomatic Body accredited to his Court. The ceremony was held in the famous White Hall of the Winter Palace. Addressing the Japanese Minister, the Czar emphasised the high value which he placed upon friendly relations with Japan, not only at the present time, but in the future. He expressed his unshaken hope that a settlement satisfactory to both nations would be arranged. Subsequently, speaking to the assembled diplomatists in general, his Majesty said, "I desire and intend to do all in my power to maintain peace in the Far East."

### Disaster at Bloemfontein.

A serious disaster was reported from Bloemfontein on Monday. Owing to continuous rain on Saturday and Sunday the town was flooded and the low-lying portion of it submerged. No fewer than 176 buildings were totally or partially destroyed; between 20 and 30 lives were lost, and 300 persons rendered homeless. The King has sent a message of sympathy for the sufferers, and the Lord Mayor of London, while not formally opening a Mansion House relief fund, has consented to remit to South Africa any sums which may be forwarded to him in aid of the distress.

### The King and Queen of Korea.

The people of Korea—a country now much before the public—claim that their history goes back to the time of Thebes and Babylon, and that from them sprang the letters, the art, and the science of Japan. Three hundred years ago the wrath of the people turned against their priests Buddhism was disestablished, and now Atheism is the universal religion, although a few Koreans believe in the doctrines of Confucius. The men possess great strength, but are calmly and passively indolent, and it is no uncommon sight to see five men digging with one spade, one man holding the handle, whilst the other four tug at it with ropes. The King is as friendly to foreigners as his father showed himself the reverse, and has further united himself with the Western world through his marriage in 1896 with Miss Emily Brown, an American lady. The present Empress of Korea is the daughter of a Presbyterian missionary from Wisconsin, and was for some time lady-in-waiting to the late Empress Min, who was mysteriously done to death in 1895. Shortly after the murder, the Emperor Yi Kong gave Miss Brown the rank of Bin, or Royal Princess, and married her, and in the following year, when a son was born, she was raised, in accordance with the dynastic laws of Korea, to Imperial rank. Their son, however, is not Crown Prince, as there was a son by the late Empress, born in 1874.

### The Late Sir Henry Keppel.

The death of the Hon. Sir Henry—generally known as Harry—Keppel took place on Sunday afternoon. The gallant Admiral of the Fleet was in his 95th year, and, in spite of his great age, had enjoyed fair health until almost a fortnight ago. As lately as December 14th he was received by the King at Buckingham Palace and remained to luncheon with his Majesty. Sir Henry Keppel, who was fourth son of the fourth Earl of Albemarle and brother of the fifth and sixth holders of the title, entered the Royal Navy before he was 15, and attained the rank of commander when he was 24. In 1837 he became Port Captain, and in 1841 he sailed in command of the *Dido* for Chinese waters, where the struggle had begun with the Celestial Government which ended in the treaty of Nankin. He subsequently distinguished himself in the Far East, and was rewarded with the Knight Commandership of the Bath. He became Rear Admiral in 1857, Groom-in-Waiting to Queen Victoria in 1859, Commander-in-Chief at Cape of Good Hope in 1860, and Commander-in-Chief in China in 1869. On August 5th, 1875, he was promoted to the rank of Admiral of the Fleet and on March 9th, 1878, was appointed first and principal Naval Aide-de-Camp to Queen Victoria. He retired on June 15th, 1879, and has since been a familiar figure in society, and has been recognised as, in a special degree, a favourite and friend of the King. In 1902 he was appointed one of the first members of the Order of Merit.

### The Somaliland Expedition.

Since General Egerton's dispatch recording the defeat of the Mullah's forces in Somaliland was received it has been officially intimated that Captain the Hon. T. Lister, elder son and heir of Lord Ribblesdale, who was signified missing, was killed on January 10th. Under date of January 16th General Egerton reports that all the wounded in the recent battle are doing well, and that the health of the troops is excellent. The loss of the enemy is now estimated to exceed 1,200. It has also been announced that the King has conferred the decoration of the Victoria Cross on Captain and Brevet Major J. E. Gough, Rifle Brigade—the Prince Consort's Own. The facts are very interesting. During the actions at Daratoleh on April 23rd last Major Gough assisted Captains Walker and Rolland in carrying back the late Captain Bruce, who had been mortally wounded, and preventing that officer from falling into the hands of the enemy. Captains Walker and Rolland had already been awarded the Victoria Cross for their gallantry on the occasion, at the suggestion of Major Gough, who was in command of the column, but he made no mention of his own conduct, which has only recently been brought to notice. Major Gough has also been promoted to the brevet rank of lieutenant-colonel in recognition of his services.

### The Curfew Bell for Dogs.

On Saturday Lord Onslow, President of the Board of Agriculture, speaking to a number of farmers at Newcastle-on-Tyne, said that it might not be disadvantageous if local authorities were empowered to ring curfew on dogs, and if the Board of Agriculture were to enact that no dog should be allowed at large after dark. His suggestions were received with much favour, and it is anticipated that he will propose legislation on these lines.

### A Book and its Story.

### THE STORY OF AN ADOPTED CHILD.*

In "Her Own People" Mrs. Croker tells with much vivacity the story of Verona Chandos, the niece and adopted daughter of a wealthy widow.

Verona had no recollection of any other relative except her aunt, Madame de Godez. We read, "Together they had led a wandering life. Madame had surely some gipsy blood in her veins and was not averse to the idea. She drifted about the Continent from one fashionable hotel to another, with a retinue of servants, tons of luggage, a parrot, a poodle, and a child. This was all very well for the parrot and the poodle, but for the child it was another affair. Her education was of a peculiar description, and undoubtedly resembled a meal where the sweets are served first." But a sudden and complete change came, in the child's life when, through the intervention of a friend she was sent to England to be educated with a girl of her own age. At Halstead Manor she lived as one of the family for nine happy years, until she was seventeen. Madame de Godez did not lose sight of her protégée. Every season she came to England and claimed her for the holidays, and on seeing her again and the remarkable promise of beauty which was visible in Verona at this age, she decided that "life was no longer possible without her adored child." So the happy schooldays at Halstead came to an end. Verona was launched on the tide of the fashionable world, and accompanied Madame de Godez to Homburg. Here there is a graphic description of the people frequenting the spa, and some clever character sketches, drawn with the quick, vivid touch that the author knows so well how to use, make these among the best chapters in the book. Verona had an air of personal distinction which was lacking in her chaperon, who was described by an onlooker as "Madame de Gaudy, the squat old woman with a dog, who reminds one of a toad with her chinless mouth, dark mottled face, and bright black eyes." Madame was reputed to be of uncertain origin. Her friends said she was of old Portuguese descent, her enemies, that she was a half-caste Portuguese. They were both in error. She alone kept the secret of her origin and her name. She was the widow of James Gowdy, a Scotchman, who had made a fortune in indigo, and she had changed the name Gowdy to de Godez after his death, as being more aristocratic and less suggestive of the humble stock from which her husband came.

On one occasion Verona had inquired, as she had frequently done before, about her parents. Madame de Godez had not until now replied definitely, but Verona was so insistent that she yielded at last, and said, "Your father and mother are both alive—in India." "My father and mother! Oh, thank God! Auntie, isn't it wonderful?" "No—ah! there is nothing wonderful at all," retorted Madame de Godez. "I knew the family; they were hard up, they had debts, and children, and as I was leaving India a widow, alone, I offered to take you to be my own daughter, and never to see them again——." "And they agreed?" exclaimed the girl, and her words were faint and tremulous. "Why, of course; it was a fine bargain for them. Oh, you were a pretty child! Just like an angel on a Christmas card." Further questioning elicited the fact that Verona's father was "a gentleman of old, old family—he looks like a duke. He was in the army, but he was hard up, and had to leave it. He is in a civil post." "And my mother?" Verona's lips dwelt lingeringly on the word. "Oh, she is not much! She is not a friend of mine. No, no, I do not like her; but she was once a beauty." "Have I any brothers and sisters?" pursued Verona. "It does not matter, for you will never see them again," replied the old lady, who was obviously disturbed and displeased. "You will never go to India; make yourself easy about that."

This phase of the heroine's life is brought to an abrupt conclusion by the sudden death of Madame de Godez. Madame had a superstitious fear of arranging her affairs. She had made three wills, all of which had been destroyed. At the earnest solicitation of her lawyer she had permitted a fourth to be drawn up; it was awaiting her signature when she was seized with a fit of apoplexy and died. Had her signature been added Verona would then have come into a fortune of fifteen thousand a year; as it was, she was left penniless. The fortune accumulated by her late uncle would now go to his relations, "Scotch farmer folk."

Now Madame was dead Verona, left penniless, turned instinctively to her own people. The Scotch heirs had been communicated with, and had arrived in London to claim their fortune. "The widow in decent black, her sons with clever Scotch faces and hands of hardworking men, clad in homespun and embarrassment, the daughter gay and complacent, with sparkling eyes and red cheeks, arrayed in a sailor hat and a gown of hunting tartan." After the funeral of her aunt Verona received a visit from the Gowdy family. Her appearance was so striking that "Mrs. Gowdy forgot herself so far as to drop a half curtsey; but Maggie never moved, merely sat and stared impassively. What was it, she wondered, that made this girl so different from herself? Her low voice, her long white throat, the delicacy of her hands, the natural dignity of her movements! Miss Chandos had something she could never possess, and that never could be taken from her!" Verona had got her parents' address, and written a letter to her mother telling her of her change of circumstances, and concluding with the following words: "I would start off at once, dearest mother, so anxious am I to see you, but Mrs. Melville advised me to wait for a reply to this letter, and also until the monsoon has broken. I am counting the very days till we meet. You will spare a little love for me, will you not?"

In reply Verona received a brief but not unkindly letter from her father, adding that he regretted the death of Madame de Godez, and also that she had made no provision for Verona. No allusion was made to her mother except in the postscript, where he wrote, "your mother sends her love." Verona would be "welcomed upon her arrival in India into what was, after all, her real home." The letter was a little disappointing to Verona, but after all it was a letter from her father, and in her loneliness she looked eagerly forward to joining the home party.

Three weeks later Verona sailed for India, and upon her arrival at Rajahpore she looked out anxiously for her own people. "Surely they were somewhere among this crowd. At last the scene began to clear, and her attention was attracted by one solitary figure. His features were finely cut, he wore a grizzled moustache, but the face was marked by the indefinable expression presented by life's failures." This was her father! Poor Verona has many disillusionments in coming among her own people, and many crushing surprises. But after all, as they were not really "her own people," we forget the miseries, and rejoice in the happier days which come to her when she joins, before long, those on whom she had the claim of kindred.

* "Her Own People." By B. M. Croker. (Hurst and Blackett. 6s.)

### Notes and Queries.

**FOR REGULATIONS SEE PAGE 135.**

*Dispensing.*

(142) I am thinking of taking up dispensing. Will you kindly give me the address of a school in London where I could be quickly taught, and where the course is not too expensive.—*A. E.*

You will find full information in the course of articles which appeared in the *Nursing Section* of THE HOSPITAL in the issues of August 29th, September 5th, 12th, and 19th last.

*Nurses' Hostel.*

(143) Can you kindly tell me where I could stay in London for a week or a fortnight, either at a club or hostel?—*Inquirer.*

At the Nurses' Hostel, Francis Street, Tottenham Court Road, W.C., or St. Andrew's House, Mortimer Street, W.

*Visiting Nurse.*

(144) I am desirous of getting visiting nursing amongst paying patients. Will you kindly tell me what I ought to charge—by the hour, or per visit, or per week?—*H. P.*

Miss Thomas, the nurse of the Marylebone Daily Visiting Nursing Association, 126 Seymour Place, Marylebone, W., would doubtless be good enough to give you advice. The usual charge is from 3s. 6d. per visit. You must arrange your scale of fees according to the amount of work you can get and the locality.

*"Snowball" Letters.*

(145) I have seen some "snowball" letters to the effect that if a million used stamps are sent to Holyede Mills before a date set by the Government, a friend has promised to build a new children's ward to a hospital near Sydney, New South Wales. I should be obliged if you will give me more particulars.—*M. M. B.*

We have not heard of such a scheme and think that it is highly improbable.

*Uniform.*

(146) Will you kindly tell me if a nurse, who has not had quite a year's training, although she has had 12 years' experience, is entitled to wear out-door uniform?—*Nurse H.*

Certainly.

*Maternity.*

(147) Will you kindly tell me if there is a maternity home or hospital where I could obtain maternity training in return for my services?—*Nurse R. L.*

As you are a nurse, possibly you might obtain free training in return for a sufficiently long period of service at a private institution.

*Abroad.*

(148) Kindly let me know where to write in order to get a post in the (1) Anglo-American Home at Rome, or (2) Cairo, or (3) Davos Dorf, etc.—*Marie O.*

(1) Write to the Matron, 265 Via Nomentana. (2) At Cairo the nursing homes are run privately, and we do not give particulars of such. (3) The Davos Invalid Home, Davos Dorf, Switzerland.

Will you kindly tell me the addresses of English nursing institutions at Cairo?—*B. B. F.*

See reply to *Marie O.*

*Home.*

(149) Can you tell me of a home where a strong girl of fifteen, who is mentally deficient, could be received and educated? She is pretty, has a bright happy disposition, and shows an aptitude for music. Her father could afford to pay a reasonable sum for her maintenance.—*Chelmsford.*

Consult the Secretary of the National Association for Promoting the Welfare of the Feeble-minded, 53 Victoria Street, S.W.

**Important Nursing Textbooks.**

"The Nursing Profession: How and where to Train." 2s. net; 2s. 4d. post free.

"A Handbook for Nurses." By Dr. J. K. Watson. 5s. net; 5s. 4d. post free.

"Practical Guide to Surgical Bandaging and Dressings." By Wm. Johnson Smith, F.R.C.S. 2s. post free.

"The Nurses' Dictionary of Medical Terms and Nursing Treatment." By Honnor Morten. 2s. post free.

"The Human Body: its Personal Hygiene and Practical Physiology." By B. P. Colton. 5s. post free.

"Art of Feeding the Invalid." (Popular Edition). 1s. 6d. post free.

"On Preparation for Operation in Private Houses. By Stanhope Bishop, F.R.C.S. 6d. post free

### For Reading to the Sick.

THANKFULNESS.

GRAVE on thy heart each past "red-letter day"!
Forget not all the sunshine of the way
By which the Lord hath led thee; answered prayers,
And joys unasked, strange blessings, lifted cares,
Grand promise-echoes! Thus thy life shall be
One record of His love and faithfulness to thee.

*F. R. Havergal.*

Let us thank God for every little pleasure, every little amusement, recreation, or delight. For they are all part of that "oil of gladness" which makes the machine of life go smoothly; they all go to form that fruit of the Spirit which is "Joy," which pervades the harmonious working of life.

*Canon Newbolt.*

Impressionable people are apt to experience seasons of very great loneliness and desolation; and then there is no remedy, save in striving to unite all such trials with His Who trod the winepress alone, Who was so "sorely wounded in the house of His friends."

It is very hard to some natures to be always cheerful, especially to those sensitive temperaments which rise and fall, barometer-like, with the influences of sunshine and east wind, whether material or moral! Yet this sensitiveness is not a fault, if struggled against; rather an additional means of seeking and serving God.

Rather dwell upon the thought of God than of yourself. Take it for granted that you are all-weak, and He all-powerful; that you will fall, and He lift you up. All that uplifts and comforts you is God's doing; think of yourself as a block of marble being hewn and chiselled by Him, and say to yourself, that even this cloud, icy and dark as it now is, will pass away, and the sunshine of His conscious Love will again gladden you.—*S. Lear.*

We have to be persuaded of the all-absorbing interest of our heavenly Father in our concerns, that He loves us individually and watches over and provides for us, that we are of much value to Him, and must go to Him for all we need. In our better moments we realise our dependence and crave for help from others; we look to friends for kindness and sympathy, and lean upon them in our hour of need; but we know that they may be taken from us, and that they, too, need help themselves. We must have God, who alone is all-sufficing, who never can change or fail, "for no other shall be accounted of in comparison with Him." He needs nothing Himself, and is able to be all in all to others.—*Anon.*

Drop thy still dews of quietness,
Till all our strivings cease;
Take from our souls the strain and stress,
And let our ordered lives confess
The beauty of Thy peace.

*J. G. Whittier.*

